# Association of Anemia Severity with Distinct Microbial and Inflammatory Signatures in Patients Receiving Vancomycin

**DOI:** 10.3390/healthcare14101417

**Published:** 2026-05-21

**Authors:** Mohammad A. Alfhili, Sahar A. Alazmi, Jawaher Alsughayyir

**Affiliations:** Department of Clinical Laboratory Sciences, College of Applied Medical Sciences, King Saud University, Riyadh 12372, Saudi Arabia

**Keywords:** anemia, vancomycin, biomarker, inflammation, ICU, sepsis

## Abstract

Background: Anemia is a pervasive public health issue that is both a risk factor and a consequence of infection. This study aims to determine the prevalence and correlates of anemia in adults receiving vancomycin (VAN). Methods: A retrospective cross-sectional analysis of clinical data was undertaken for 299 patients treated with VAN at a tertiary care hospital from January 2024 to February 2025. Subjects were stratified by anemia severity into non-anemic, mild, moderate, and severe groups. Frequency was examined using the chi-squared test, medians by Kruskal–Wallis test, monotonic relations by Spearman’s correlation, and independent predictors using regression models. Results: Anemia was extremely prevalent in 90% of patients, mostly at a moderate level, and a weak positive correlation was observed between anemia severity and VAN trough levels, ICU admission, kidney disease, abnormal liver markers, and inflammatory indices. Microbial isolates were differentially enriched across anemia categories with *K. pneumoniae*, *E. coli*, and MRSA peaking in mild anemia and sharply declining in moderate and severe cases. Anemia severity was differentially correlated with *P. aeruginosa*, creatinine, hypertension, liver disease, albumin, platelets, and derived indices. In adjusted analysis, albumin, age, gender, platelet–neutrophil ratio, kidney disease, ICU admission, and MATH-1SD were independent predictors of anemia. A diagnostic model for anemia based on multiple markers was developed with an accuracy of 77%. Conclusions: Anemia is alarmingly very common in VAN-treated patients with distinct microbial and inflammatory signatures across severity groups, which highlights the need for experimental and longitudinal studies elucidating its pathophysiological mechanisms and clinical implications.

## 1. Introduction

Anemia is a condition characterized by the compromised ability of red blood cells (RBCs) to deliver oxygen. This could arise because of reduced RBC numbers or function due to diminished hemoglobin. Affecting 25% of the global population [1], anemia prevalence has disproportionately increased over the past three decades in countries with a low sociodemographic index [2]. Female gender, older age, low socioeconomic status, frequent blood donation, alcoholism, and some medications constitute the major risk factors of anemia. These factors predispose patients to the wide spectrum of etiologies implicated in various types of anemia including nutritional deficiencies, chronic disease, blood loss, bone marrow suppression, and RBC death. Typical presentation includes fatigue, dyspnea, pallor, and tachycardia, and management depends on treating the underlying cause, often through supplements, erythropoiesis-stimulating agents, immunosuppressants, splenectomy, RBC transfusion, and bone marrow transplantation [3].

Several lines of evidence ascertain that anemia is both a risk factor and a consequence of infection and treatment. Anemia is associated with subclinical infection [4], invasive bacterial infections [5], enteropathogens [6,7,8], typhoidal fever in glucose-6-phosphate dehydrogenase patients [9], sepsis and mortality in sickle cell disease [10], and morbidity and mortality in children [11]. Anemia is also a risk factor for tuberculosis (TB) infection and complications [12,13], whereas *Helicobacter pylori* predisposes to anemia [14]. Mechanisms underlying infection-related anemia include bone marrow infiltration [9], cytokine production, particularly interleukin-6 and tumor necrosis factor α, hepcidin modulation [15], and nuclear factor kappa-light-chain-enhancer of activated B cell signaling [16]. Recently, the role of microbiome ecology has been recognized as a contributing factor driving anemia in diverse populations [17,18,19], further confirming the integral role of bacterial community composition in the pathophysiology of anemia.

Vancomycin (VAN) is a glycopeptide inhibitor of cell wall synthesis which makes it effective against Gram-positive bacteria. Administered by intravenous infusion for systemic infections and orally for *Clostridium difficile*, VAN is excreted by the kidneys, and dosing is therefore adjusted based on renal function. Due to its narrow therapeutic index, VAN requires continuous monitoring by serial measurements of trough levels. Beside nephrotoxicity, ototoxicity, and red man syndrome, anemia is a rare and under-recognized adverse effect of VAN treatment [20]. Despite their scarcity, previous reports have shown that VAN causes a false-positive direct antiglobulin test with anti-IgG and a host of antiglobulin reagents, as well as RBC aggregation through its polycationic nature and membrane protein binding [21]. A positive indirect antiglobulin test in the presence of VAN has also been documented [22] and corroborated by a later report [23]. Patients often exhibit severe anemia with hemoglobin levels as low as 4.2 g/dL which improve after VAN discontinuation [20,23,24,25].

To date, very little is known about anemia in the setting of VAN and infection, and diagnostic and therapeutic precision remains a challenge. The objective of the current study is to explore the prevalence and clinical correlates of anemia in adults receiving VAN which may clarify contributing factors and improve clinical management of anemia in the setting of infection.

## 2. Patients and Methods

### 2.1. Study Population and Setting

This study design complies with the STROBE guidelines for observational studies. This retrospective, cross-sectional study was conducted on 299 adult patients during VAN treatment at King Khalid University Hospital, Riyadh, Saudi Arabia from January 2024 to February 2025 following ethical approval from the IRB Committee of King Saud University (E-25-9553). In line with the Declaration of Helsinki, patient identifiers were anonymized to preserve confidentiality. All adult patients (≥18 years) who received VAN during the study period were included. Patients were excluded if aged <18 years or if they had missing or incomplete hemoglobin or laboratory data required for calculation of inflammatory ratios or analysis of microbiological profiles. Systematic exclusion based on pre-existing anemia, myelosuppressive medications, or transfusions was not feasible due to the unavailability of these factors in the hospital registry, although they are acknowledged as potential sources of confounding. Given the retrospective and real-world nature of this study, no a priori sample size calculation was performed, and this study included all eligible patients representing a consecutive real-world population. Demographic and clinical variables including age, gender, blood pressure (BP), body mass index, comorbidities, and intensive care unit (ICU) admission status were retrieved from hospital records.

### 2.2. Laboratory Parameters

Hematology and chemistry tests were carried out along with VAN trough level determination as per the Saudi Ministry of Health guidelines (https://www.moh.gov.sa/Ministry/MediaCenter/Publications/Documents/Protocol-002.pdf (accessed on 10 August 2025)), using XN-2000 (Sysmex, Hyogo, Japan) and Cobas 8100 (Roche, Indianapolis, IN, USA) automated analyzers, respectively. A wide assortment of emerging ratios and indices derived from routine hematology and chemistry tests were investigated for their association with anemia severity. Collectively, these parameters serve as surrogate markers of systemic inflammation, nutritional status, and organ function, making them relevant to both infection and anemia pathophysiology. These markers include the prognostic nutritional index (PNI), hemoglobin–albumin–lymphocyte–platelet index (HALP), systemic inflammation response index (SIRI), neutrophil lymphocyte ratio (NLR), derived neutrophil–lymphocyte ratio (dNLR), coefficient of variation in red cell distribution width (RDW-CV)–platelet ratio (RPR), lymphocyte–monocyte ratio (LMR), platelet–neutrophil ratio (PNR), fibrosis-4 index (FIB-4), aggregate index of systemic inflammation (AISI), gamma-glutamyl transferase (GGT)–platelet ratio (GPR), C-reactive protein (CRP)–albumin–lymphocyte index (CALLY), systemic inflammatory index (SII), mean platelet volume–lymphocyte ratio (MPVLR), platelet–creatinine ratio (PCR), aspartate transaminase–platelet ratio index (AST APRI), CRP/albumin ratio (CAR), platelet–monocyte ratio (PMR), platelet–lymphocyte ratio (PLR), alanine transaminase–platelet ratio index (ALT APRI), eosinophil–lymphocyte ratio (ELR), LDH–albumin ratio (LAR), platelet–albumin ratio (PAR), lymphocyte–CRP ratio, CRP–albumin ratio, and erythrocyte sedimentation rate (ESR).

Microbial isolates and resistance patterns can be accessed through our previous publication [26]. The present study utilizes the same cohort but addresses distinct clinical questions. Using WHO criteria [27], subjects were stratified based on their anemia status according to hemoglobin levels (g/dL) into non-anemic (≥13.0 for males; ≥12.0 for females), mild (11.0–12.9 for males; 11.0–11.9 for females), moderate (8.0–10.9), and severe (<8.0) groups.

### 2.3. Statistical Analysis

Prism 9.0 (GraphPad, San Diego, CA, USA) and Excel 2016 (Microsoft, Redmond, WA, USA) were used for all statistics with a significance threshold of *p* < 0.05. Skewed variables failed the Shapiro–Wilk test and were therefore analyzed by the Kruskal–Wallis test, followed by Dunn’s test with adjustment by Bonferroni test and by Spearman rank correlation analysis. Given the number of correlated indices tested against the same outcome, false discovery rate (FDR) correction using the Benjamini–Hochberg method was applied to mitigate the risk of spurious associations (type I error). Proportions were compared by the chi-squared test, and independent predictors were identified using regression models. Correlation was performed with ordinal anemia severity as the outcome using a single independent variable at a time (bivariate analysis), while log-transformed hemoglobin was used as the dependent variable in simple and multiple linear regression models. The discriminatory potential of key variables to identify anemic subjects was assessed using receiver operating characteristic (ROC) curve analysis with the area under the curve (AUC) reported. The Hosmer–Lemeshow method was used to evaluate model calibration. Internal validation was performed using bootstrap resampling in which samples of equal size were drawn with replacement to calculate the optimism-corrected AUC across resamples.

## 3. Results

The non-anemic group was substantially smaller (*n* = 30) than the anemic groups, reflecting the high prevalence of anemia in this cohort. This group did not differ significantly in age compared to the other groups (Table 1). However, non-anemics had significantly higher diastolic blood pressure, Ca^2+^, albumin, ALT, RBCs, hematocrit, hemoglobin, and monocytes (Table 2) and significantly lower RDW-CV and MATH-1SD (Table 2). Moreover, PNI, HALP, R ratio, SIRI, RPR, and CALLY were significantly elevated in non-anemics compared to anemic patients (Table 3).

**Figure 1:**
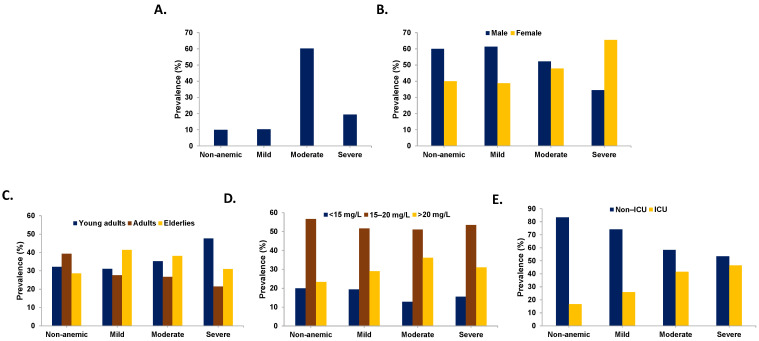
Prevalence of anemia. Proportions of patients in each anemia category are shown in (**A**) the whole cohort and after stratification by (**B**) gender, (**C**) age group, (**D**) vancomycin trough levels, and (**E**) ICU requirement. Chi-squared test was used for statistical comparison of frequencies.

As seen in Figure 1A, anemia was extremely common with a prevalence of 89.97%, the majority of which (60.2%) were moderate followed by severe (19.39%) and mild cases (10.36%). Stratified by gender (Figure 1B), every anemia stage was dominated by males except severe cases in which females were 65.52%. The effect of gender on anemia distribution was significant as revealed by the chi-squared test (*X*^2^ = 8.69, *p* = 0.0337). However, this was not the case when patients were divided by age group (*X*^2^ = 6.10, *p* = 0.4124) or trough levels (*X*^2^ = 3.27, *p* = 0.7744) as revealed in Figure 1C,D, respectively. Interestingly, Figure 1E shows that there was a significantly increasing trend in the proportion of anemic patients in the ICU (*X*^2^ = 10.50, *p* = 0.0147) with critical illness, constituting 16.67%, 25.81%, 41.67%, and 46.55% of non-anemic, mild, moderate, and severe cases, respectively.

Table 1 shows that BP was significantly altered according to anemia stage and that patients with renal disease were significantly enriched as anemia worsened (*p* = 0.0036). In Table 2, Ca^2+^, albumin, alanine transaminase, GGT, direct bilirubin, RDW-CV, MATH-1SD, and white blood cells were significantly different among the four groups. Alarmingly, despite the high prevalence of anemia, only a little less than 10% of patients had biochemical anemia tests, including iron studies, B_12_, and folic acid, available. Distinct inflammatory markers were also modulated according to anemia stage (Table 3). These include PNI, HALP index, R ratio, SIRI, NLR, RPR, LMR, PNR, FIB-4, AISI, GPR, and CALLY index.

**Figure 2:**
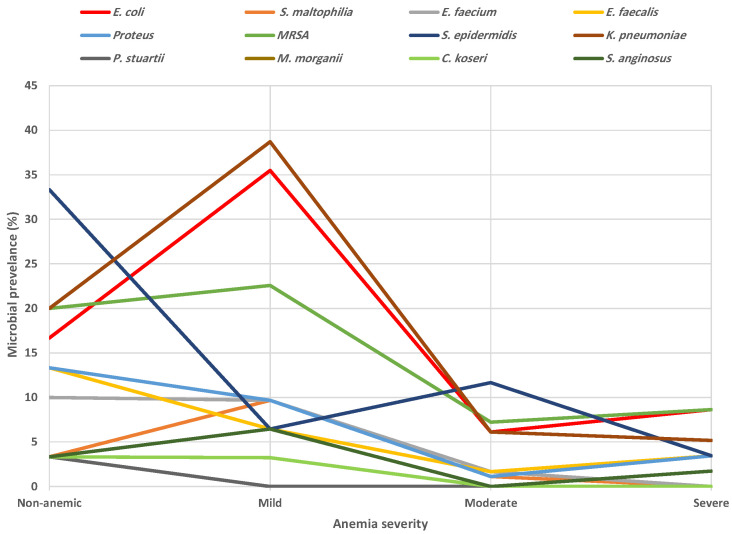
Distribution of isolated organisms across anemia severity categories. Proportions of significantly enriched pathogens per anemia group as analyzed by the chi-squared test are displayed.

Analysis of positive cultures by chi-squared test for trend (Figure 2) revealed significant enrichment of distinct organisms according to anemia severity. Of note, positive isolates in the non-anemic group indicate that VAN use was primarily therapeutic rather than prophylactic. In particular, *Klebsiella pneumoniae* (*X*^2^ = 33.98, *p* < 0.0001) and *Escherichia coli* (*X*^2^ = 7.92, *p* = 0.0049) demonstrated a clear peak in mild cases (38.71% and 35.48%, respectively) before sharply declining as anemia worsened. Methicillin-resistant *Staphylococcus aureus* (MRSA; *X*^2^ = 10.08, *p* = 0.0179) followed a similar pattern although the elevation was less pronounced (22.58%) while the patterns exhibited by *Stenotrophomonas maltophilia* (*X*^2^ = 4.66, *p* = 0.0309) and *Streptococcus anginosus* (*X*^2^ = 9.55, *p* = 0.0228) were modest. Likewise, *Enterococcus faecium* (*X*^2^ = 12.65, *p* = 0.0054), *Enterococcus faecalis* (*X*^2^ = 10.63, *p* = 0.0139), and *Proteus* (*X*^2^ = 14.40, *p* = 0.0024) showed a decreasing trend as anemia severity increased. An inverse pattern was observed for *Staphylococcus epidermidis* (*X*^2^ = 18.23, *p* = 0.0004), which decreased from 33.33% in non-anemics to as low as 3.45% in severe cases.

Table 4 shows that anemia severity correlates with traditional and composite inflammatory indices along with hepatic and renal markers as shown by Spearman rank correlation. Importantly, all reported associations remained statistically significant after FDR correction, suggesting that findings are unlikely to be attributed to multiple testing alone. Additionally, regression analysis (Table 5) revealed several independent predictors of anemia. Age, albumin, and PNR were not significantly associated with hemoglobin in the univariate analysis but emerged as independent predictors of anemia severity after adjustment. In contrast, gender, renal disease, ICU status, and MATH-1SD persisted even after adjustment.

**Figure 3:**
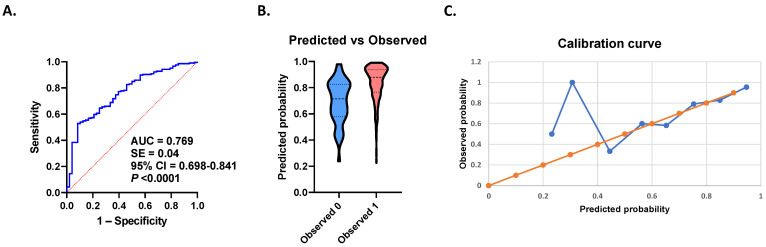
Anemia diagnostic model. (**A**) Receiver operating characteristic curve of anemia discrimination based on age, ICU admission, hypertension, and NLR. (**B**) Distribution of predicted probabilities according to observed anemia status. (**C**) Calibration curve comparing predicted and observed probabilities of anemia across probability bins.

In Figure 3A, the diagnostic performance of key variables to distinguish anemic from non-anemic patients undergoing VAN therapy is shown in an ROC curve. Variable selection was based on clinical and statistical significance. At the predefined classification threshold (0.5), the overall accuracy of the model was 77% (*p* < 0.0001) with a positive predictive value (PPV) of 83%, a negative predictive value (NPV) of 58.33%, sensitivity of 97.56%, and specificity of 14.58% collectively based on age (*p* = 0.0207), ICU admission (*p* = 0.0008), hypertension (*p* = 0.0189), and NLR (*p* = 0.0024). Internal validation revealed lower but consistent performance, with a bootstrap optimism-corrected AUC of 0.699, indicating modest overfitting. The predicted probabilities plot (Figure 3B) shows there is separation between non-anemic and anemic subjects albeit with substantial overlap, which reflects the model’s high sensitivity but limited specificity in a high-prevalence setting. Additionally, the calibration curve based on deciles of predicted probability (Figure 3C) showed deviations from the ideal diagonal at lower ranges due to the smaller sample sizes within individual bins, while calibration was more consistent at moderate-to-high ranges. Such instability in low-frequency bins is a limitation of decile-based calibration in imbalanced subgroups rather than consistent model miscalibration.

## 4. Discussion

Anemia is a severely understudied complication during infection and VAN therapy, and this work is the first to identify microbial and inflammatory correlates of anemia severity in VAN-treated adults. Although the exact etiologies of anemia in our patients cannot be ascertained, the current findings underscore the need for a better understanding of drug- and infection-related mechanisms, which will be invaluable for prevention, diagnosis, and risk stratification. Importantly, the current findings must be interpreted as observational associations within a VAN-treated cohort rather than causal effects attributable to VAN exposure due to the absence of a comparator cohort. Instead, anemia in this population appears to be multifactorial, influenced by infection, inflammation, comorbidities, and iatrogenic interventions.

The literature on the association between VAN exposure, inflammation, and bacterial infections is scarce, and we were therefore prompted to contextualize our findings using evidence from closely related and extensively studied infections as analog models. In these conditions, the pathophysiological mechanisms underlying anemia and immune dysregulation are similar to those observed in bacterial infections, although they do not constitute a direct equivalence. Future longitudinal studies examining the causal relationship among hematinic, inflammatory, and bacterial profiles under VAN exposure are likely to validate these observations.

A striking finding in this study is the high prevalence of anemic patients among subjects on VAN therapy, reaching 90%, the majority of which had moderate severity (Figure 1A). This is in agreement with previous studies on anemia in infected individuals. Lai et al. [28] reported that anemia in human immunodeficiency virus (HIV) patients with TB was significantly more common than those without TB, and a similar finding was also observed for those with and without *Penicillium marneffei*. In that study, anemia prevalence also correlated with viral load. In an Iranian cohort [29], it was reported that anemia was present in 71% of HIV patients and was associated with mortality. Importantly, the lack of a comprehensive anemia workup in the majority of patients in this study highlights the need to incorporate iron homeostasis markers, B_12_, and folic acid in routine management to improve patient outcomes.

Although anemia primarily affects children and females, which is consistent with our results (Figure 1B), several studies reported varied patterns. For example, in Bahrain, 71.6% of elderly hospitalized patients were anemic and males were more affected [30]. Anemia was also more common in male coronavirus disease 19 (COVID-19) patients [31]. Nonetheless, in HIV patients, age and gender did not significantly predict anemia in adjusted analysis [28]. In our cohort, age and gender were independent predictors of anemia severity (Table 5), which agrees with earlier findings [32,33]. However, the positive association between age and hemoglobin level reported herein is similar to that observed for females beyond reproductive years [34] and may reflect the increased requirement for transfusion in older patients or the potential confounding effect of medication or supplement intake, although the latter has previously been questioned [35].

The current study also revealed that anemia was significantly more common in the ICU (Figure 1E), which correlated with (Table 4) and predicted (Table 5) severity. This is in line with observations in sepsis [36] and COVID-19 [1] patients. During critical illness, anemia may develop due to blood loss, myelosuppression, nutritional deficiencies, coagulopathies, and RBC death [37]. Indeed, renal disease was significantly more common in anemic subjects (Table 1), increased progressively with severity (Table 4), and resulted in a decrease by 7.7% of hemoglobin (Table 5). In fact, nephrotoxicity is a major side effect of VAN administration and could similarly contribute to the observed high prevalence of anemia in this cohort. Nonetheless, such a temporal relationship requires longitudinal studies to confirm its existence. This is significant because renal insufficiency is invariably complicated by anemia due to perturbed erythropoietin production, which predisposes patients to poor outcomes and increased mortality. Compromised nutrient utilization and hemorrhage are also other recognized mechanisms underlying anemia in renal disease [38].

In this regard, the PCR was negatively correlated with anemia severity (Table 4), and a 10% decrease led to an 8% fall in hemoglobin (Table 5). Congruently, creatinine was positively correlated with severity while platelets and related indices were negatively correlated (Table 4). Although poorly studied, lower PCR has been shown to predict mortality in aortic dissection [39] and cirrhotic [40] patients. Thrombocytopenia is a side effect of VAN [41] and could arise secondary to kidney disease [42] and a host of infections [43]. Collectively, the PCR seems to be a novel composite marker that captures coagulopathic, hepatic, and renal complications during infection, which deserves further investigation.

Variations in liver markers were consistently associated with anemia severity in this patient cohort. In particular, albumin levels were proportional to hemoglobin decrease, while direct bilirubin, alkaline phosphatase, and GGT increased as anemia worsened (Table 2 and Table 3). Beside VAN-induced hepatotoxicity, which causes transient elevations in liver enzymes [44], hypoalbuminemia is a recognized risk factor for various bacterial and viral infections, and albumin infusion has shown great promise in clinical trials to complement antimicrobial therapy [45], further cementing its importance as an orchestrator of infection outcomes. Moreover, being an independent predictor of anemia severity (Table 5), hypoalbuminemia and anemia are known comorbidities in opportunistic infections [46] and significantly increase all-cause mortality in nontuberculous mycobacterial lung disease [47]. In this regard, albumin, along with age and PNR, were significantly associated with anemia severity after multivariable adjustment and their absence in the univariate analysis seems to be due to confounding, which is common in heterogenous patient populations, especially with small effect sizes.

Along those lines, this work identified multiple liver-derived ratios and indices, including AST APRI, GPR, CALLY, FIB-4, and R ratio, significantly modulated by anemia status (Table 3 and Table 4). AST APRI and GPR may reflect diminished platelets often seen in infections and have recently been reported as predictors of various infections [48,49,50,51,52]. CALLY is another emerging marker of nutrition and inflammation that specifically differentiated severe from non-anemic cases (Table 3), which indicates it might not be as sensitive as other markers like LMR and PNR. Nonetheless, previous studies have shown that CALLY is a predictor of mortality in sepsis patients [53,54]. Likewise, it has been found that FIB-4 is a predictor of anemia and leukopenia in distinct populations [55,56], suggesting a potential mechanistic link between liver injury and adverse hematological events.

RDW-CV and its derived ratios, MATH-1SD and RPR, demonstrated an increasing trend with anemia (Table 2, Table 3, Table 4 and Table 5), although the latter was the least sensitive, identifying only severe cases. A general marker of inflammation, RDW-CV displayed a significant association with worse outcomes in anemic TB patients [13], treatment delay [57], and mortality in sepsis [58,59]. Our findings are in concordance with previous reports and lend support to the incorporation of red cell size in the staging of patients with various bacterial infections, pending confirmation from longitudinal studies.

Several reports have examined the crosstalk between anemia and immune dysregulation, albeit using traditional markers. For example, anemia in TB was associated with increased inflammatory cytokines and higher bacterial loads [60], and interleukin-6 demonstrated a negative correlation with hemoglobin in critically ill sepsis patients [36] and was higher in anemic COVID-19 subjects compared to non-anemics [61]. Furthermore, anemia was a risk factor for bacterial complications and mortality in HIV patients [62]. In addition to routine parameters such as CRP (Table 4), this work identifies numerous emerging inflammatory ratios and indices associated with anemia. Diminished ratios included PNI, HALP, SIRI, AISI, LMR, PNR (Table 3), and lymphocyte–CRP ratio (Table 3 and Table 5), whereas NLR and CRP–albumin ratio were elevated (Table 4). The decrease in PNI reflects hypoalbuminemia and lymphopenia (Table 2), which agrees with its reported role as a predictive marker of mortality in community-acquired bacterial pneumonia [63]. SIRI was a sensitive marker of catheter-related bloodstream infection in hemodialysis patients [64] while AISI outperformed other inflammatory markers in detecting odontogenic abscess severity [65]. As for NLR, it has recently been shown to be associated with pneumonia severity [66], infection risk in rheumatoid arthritis surgical patients [67], and mortality due to bacteremia [68]. In the current study, the increase in NLR with anemia severity is largely attributed to lymphopenia which is related to anemia and recurrent infection [69,70]. Chemotaxis, enhanced apoptosis, and myelosuppression may account for the reduced peripheral lymphocyte counts encountered during infection, but more experimental and clinical studies are needed to confirm this association.

Several bacterial pathogens were distinctly enriched in different anemia severities (Figure 2). Perhaps most important is the pattern exhibited by *E. coli* and *K. pneumoniae*, which peaked at moderate level and then dropped as anemia progressed. The pathognomonic features of hemolytic uremic syndrome, caused by Shiga toxin-producing *E. coli*, are microangiopathic anemia, renal injury, and thrombocytopenia [71], all of which are consistent with our findings. Recently, *K. pneumoniae* has been shown to recruit macrophages to acquire iron [72], suggesting that iron deprivation may be a possible mechanism behind the observed anemia in our cohort. Furthermore, anemia was an independent risk factor for mortality due to bloodstream infections by MRSA [73], and a recent review found evidence of higher mortality in MRSA patients treated with VAN in comparison to those who received linezolid [74]. However, linezolid has long been known as a myelosuppressive agent that causes anemia [75], which might not be advantageous to VAN in that regard.

In correlation analysis, *Pseudomonas aeruginosa* was the sole pathogen that was weakly associated with the severity of anemia (Table 4). *P. aeruginosa* concurrent with anemia has previously been described [76,77] and, along with *M. morganii*, *Proteus*, and *E. faecalis*/*avium*, was isolated in a confirmed case of VAN-induced anemia [20]. Notably, infection by *P. aeruginosa* is subject to regulation by nitric oxide [78], a known modulator of RBC lifespan [79]. *Enterococcus*- and *Enterobacter*-positive cultures were also observed in another confirmed case of VAN-induced anemia [24]. Therefore, experimental and longitudinal studies are likely to elucidate the underlying mechanisms linking various pathogens to anemia during VAN therapy.

Although the diagnostic model presented in Figure 3 shows an AUC of 0.769, which is the primary prevalence-independent measure of discrimination, it nevertheless exhibits very high sensitivity at the expense of specificity, which reflects the high prevalence of anemia in this cohort. As a consequence, the PPV appears elevated while the NPV remains modest, making the model best suited for identifying patients with anemia rather than excluding it. Notably, a reduction in model performance was noted upon internal validation (AUC decreasing from 0.769 to 0.699) indicating modest overfitting. As a binary classifier, it represents an exploratory and descriptive model rather than a definitive clinical instrument, and external validation in independent cohorts with more balanced prevalence is therefore required.

The current study suffers from limitations inherent to cross-sectional designs including selection bias, residual confounding, incomplete clinical variables, and the inability to determine causation. A weak, non-independent association between VAN trough levels and anemia severity did not persist following multivariable adjustment and must therefore not be considered evidence of a causal relationship. The single-center setting also limits generalizability, and the cause of anemia including VAN-induced immune hemolysis, sepsis, iron deficiency, cytokine-mediated erythropoiesis suppression, hepcidin upregulation, blood loss, or hemodilution is lacking. Reticulocyte count, Coombs test, and haptoglobin levels were not available and prevented anemia subtyping. Moreover, the comparatively small size of the non-anemic group introduces asymmetry and may increase sensitivity to outliers. Additionally, the potential effect of confounders such as admission timing, duration of VAN therapy, pre-existing anemia prior to VAN initiation, recent transfusion, medication intake, or nutritional status is unknown. Furthermore, a key limitation of the current study is the exclusion of patients who did not receive VAN, which prevented broader comparisons. It is also important to note that the differential microbial profiles observed herein might have been caused, at least in part, by sampling bias, prior antibiotic exposure, or survivorship status, which must be accounted for in future longitudinal studies. Also, although several associations reached statistical significance, the majority of correlation coefficients fall in the weak range (*ρ* < 0.3), including VAN trough levels, and therefore explain less than 4% of the variance in anemia severity. Therefore, a clear distinction between statistical and clinical significance in this cohort is particularly important since the sample size permits detection of small effect sizes that may have limited clinical utility. Notably, the limited availability of hematinic and cytokine data precludes the ability to link observed associations to specific mechanisms. As such, the current findings must be interpreted as exploratory and descriptive rather than evidence of VAN-induced effects. In contrast, our study has several strengths including a relatively large, well-characterized patient population, exploration of traditional and emerging markers, streamlined data acquisition, robust analysis, and segregation of microbial pathogens across anemia strata.

## 5. Conclusions

In conclusion, anemia was extremely prevalent in adult patients receiving VAN and was associated with inflammatory and microbial profiles. Although observational as opposed to causal, the current findings may aid in risk stratification and diagnosis of anemic states in infected patients. Future research should focus on elucidating the mechanisms underlying the observed associations, in addition to identifying the potential causal relationships governing VAN administration, anemia incidence and progression, microbial profiles, and biomarker variation.:

## Figures and Tables

**Table 1:** Patient characteristics stratified by anemia severity.

Variable	Non-Anemic (*n* = 30)	Mild (*n* = 31)	Moderate (*n* = 180)	Severe (*n* = 58)	*p* Value
Age (years)	58.50 (50.86–63.74)	61.0 (49.24–65.28)	61.50 (55.73–61.30)	55.50 (49.31–58.93)	0.4973
Gender (%)					**0.0337**
Male	60.0	61.29	52.22	34.48	
Female	40.0	38.71	47.78	65.52	
SBP (mmHg)	126.5 (115.2–131.6)	119.0 (115.2–128.5)	113.0 (110.9–117.9)	119.0 (113.3–124.6)	**0.0495**
DBP (mmHg)	70.50 (64.13–73.52)	65.00 (60.25–69.27)	60.00 (59.92–63.63)	64.00 (61.13–68.63)	**0.0213 ^#^**
Weight (kg)	73.00 (68.19–81.62)	66.60 (64.98–82.06)	70.00 (66.73–73.65)	66.50 (62.81–76.29)	0.5037
BMI	26.50 (25.51–31.25)	26.58 (25.60–32.43)	26.23 (25.53–27.88)	25.60 (24.45–29.02)	0.7054
Vancomycin dose (mg)	1250 (1043–1298)	1000 (1002–1303)	1000 (1023–1133)	1250 (1042–1275)	0.2891
Kidney disease (%)	3.33	6.45	17.22	24.14	**0.0036**
Liver disease (%)	3.45	0	6.71	4.55	0.4559
Heart disease (%)	28.57	20.69	20.86	16.67	0.6948
Diabetes mellitus (%)	39.29	37.93	34.53	30.95	0.8847
Coagulopathy (%)	3.57	0	3.60	0	0.6589
Hypertension (%)	42.86	48.28	35.97	26.19	0.2444
Cancer (%)	14.29	6.90	17.27	26.19	0.0926

Results are shown as median ± 95% confidence intervals or percentage of category total as analyzed by the Kruskal–Wallis test or chi-squared test for trend as appropriate. SBP, systolic blood pressure; DBP, diastolic blood pressure; BMI, body mass index. ^#^ Moderate vs. non-anemic. Bold *p* values are statistically significant.

**Table 2:** Comparison of laboratory parameters stratified by anemia severity.

Variable	Non-Anemic	Mild	Moderate	Severe	*p*
Vancomycin trough (mg/L)	15.05 (12.83–19.13)	17.30 (13.97–20.08)	16.30 (15.98–18.83)	14.35 (13.86–18.65)	0.6798
Anemia profile					
Iron (μM)	5.90 (3.84–14.86)	5.54 (2.76–8.32)	7.73 (4.97–10.90)	12.02 (4.10–31.99)	0.7568
TIBC (μM)	35.24 (15.36–43.80)	33.96 (21.72–44.38)	33.09 (26.04–40.78)	40.50 (34.65–41.67)	0.8611
UIBC (μM)	31.40 (0.50–37.90)	22.30 (13.40–31.20)	27.0 (17.35–32.85)	15.30 (4.30–37.80)	0.9162
Transferrin (μM)	354.4 (154.4–440.5)	341.5 (218.4–446.3)	332.8 (261.8–410.1)	407.3 (348.4–419.0)	0.8611
Transferrin saturation (%)	13.47 (10.90–96.47)	23.22 (8.12–38.31)	23.54 (16.80–31.45)	55.84 (9.28–89.38)	0.8717
Ferritin (pM)	217.0 (153.0–142.1)	119.8 (57.50–182.0)	693.0 (407.0–1618.0)	1435 (209.0–4966)	0.1750
B_12_ (pM)	538.0 (162.0–914.0)	253.0 (106.0–702.0)	894.0 (593.0–1853)	731.0 (578.0–2952)	0.2191
Glycemic control					
Glucose (mM)	6.4 (5.5–7.7)	8.0 (6.0–9.4)	6.8 (6.0–7.4)	6.8 (5.9–8.2)	0.5491
Hb_A1c_ (%)	6.5 (5.8–7.6)	7.8 (5.3–15.3)	6.5 (5.8–7.8)	6.8 (6.5–10.2)	0.3697
Renal function tests					
Creatinine (μM)	76.5 (63.0–85.0)	74.0 (65.0–92.0)	94.5 (82.0–105.0)	100.0 (69.0–131.0)	0.2937
Na^+^ (mM)	137.5 (135.6–139.1)	139.3 (136.9–141.1)	138.3 (137.3–139.2)	136.8 (134.1–139.0)	0.1388
K^+^ (mM)	4.0 (3.8–4.3)	4.2 (4.1–4.5)	4.1 (3.9–4.2)	4.1 (3.9–4.3)	0.2626
PO_4_^−^ (mM)	1.1 (0.9–1.2)	1.1 (1.0–1.3)	1.1 (1.0–1.2)	1.2 (1.0–1.3)	0.7876
HCO_3_^−^ (mM)	21.9 (18.0–23.9)	22.8 (20.2–23.5)	21.4 (20.5–22.1)	21.1 (18.2–22.3)	0.7507
CO_2_ (mM)	23.2 (21.5–24.5)	24.2 (22.8–26.0)	22.7 (21.7–23.3)	22.5 (21.3–23.6)	0.0960
Cl^−^ (mM)	100.7 (98.3–103.4)	102.8 (101.5–105.8)	102.9 (101.6–104.0)	101.1 (99.3–104.2)	0.3251
Mg^2+^ (mM)	0.9 (0.8–1.0)	0.9 (0.7–1.0)	0.8 (0.8–0.9)	0.8 (0.8–0.9)	0.3910
Ca^2+^ (mM)	2.2 (2.1–2.2)	2.2 (2.1–2.3)	2.0 (2.0–2.1)	2.0 (2.0–2.1)	**<0.0001** ^#,†,‡,¶^
Corrected Ca^2+^ (mM)	2.3 (2.2–2.3)	2.4 (2.3–2.4)	2.3 (2.2–2.3)	2.3 (2.2–2.3)	**0.0028** ^‡^
Liver function tests					
Albumin (g/L)	34.3 (31.3–36.9)	32.7 (28.5–34.4)	28.8 (27.4–29.6)	25.4 (25.0–28.3)	**<0.0001** ^#,†,‡,¶^
ALT (U/L)	31.4 (23.6–36.9)	15.8 (10.4–22.9)	20.8 (17.2–27.5)	16.9 (12.8–22.4)	**0.0067** *^,†^
AST (U/L)	26.2 (20.5–31.5)	20.2 (13.9–29.3)	27.7 (23.6–35.6)	23.9 (21.9–33.6)	0.1319
ALP (U/L)	116.5 (87.0–134.0)	97.0 (78.0–110.0)	111.5 (98.0–123.0)	118.5 (94.0–155.0)	0.1952
GGT (U/L)	76.5 (49.0–105.0)	28.0 (23.0–50.0)	52.0 (44.0–65.0)	49.5 (36.0–62.0)	**0.0173** *
Total bilirubin (μM)	6.7 (5.4–9.1)	7.6 (4.2–9.2)	8.1 (6.5–9.3)	8.9 (5.9–14.0)	0.2185
Direct bilirubin (μM)	3.0 (2.6–5.9)	3.1 (2.2–4.1)	4.4 (3.8–5.4)	5.8 (3.7–8.4)	**0.0064** ^‡,¶^
Indirect bilirubin (μM)	3.6 (2.4–4.4)	2.5 (2.1–4.5)	3.0 (2.7–3.4)	3.2 (2.3–4.1)	0.6377
Complete blood count					
RBCs (×10^6^/μL)	4.8 (4.6–5.1)	4.1 (3.9–4.4)	3.3 (3.2–3.4)	2.6 (2.5–2.7)	**<0.0001** ^#,†,‡,¶,§^
Hematocrit (%)	40.4 (39.7–41.9)	36.3 (35.5–37.2)	29.1 (28.6–29.6)	23.0 (22.3–23.7)	**<0.0001** ^#,†,‡,¶,§^
Hemoglobin (g/dL)	13.3 (13.1–13.5)	11.7 (11.4–11.8)	9.3 (9.1–9.4)	7.3 (7.2–7.5)	**<0.0001** ^#,†,‡,¶,§^
MCH (pg)	27.6 (27.1–28.2)	28.8 (27.2–29.2)	28.3 (27.7–28.6)	28.2 (27.3–29.0)	0.7169
MCHC (g/L)	324.0 (322.0–329.0)	320.0 (312.0–328.0)	320.0 (316.0–322.0)	317.0 (312.0–324.0)	0.3813
MCV (fL)	84.2 (81.7–87.0)	88.3 (84.8–90.4)	86.6 (85.2–87.9)	85.8 (84.4–89.2)	0.1638
RDW-CV (%)	13.5 (13.2–14.6)	14.8 (13.8–15.9)	16.5 (16.2–17.1)	17.9 (16.7–18.6)	**<0.0001** ^#,†,‡,¶^
MATH-1SD	11.3 (11.2–12.4)	13.2 (12.4–14.1)	14.3 (13.7–15.0)	15.8 (14.7–16.8)	**<0.0001** ^#,†,‡,¶^
WBCs (×10^3^/μL)	11.4 (8.0–13.2)	8.0 (6.5–10.9)	10.0 (9.1–10.7)	9.6 (7.0–11.5)	0.1766
Neutrophils (×10^3^/μL)	8.2 (4.8–10.7)	5.1 (3.5–7.1)	7.4 (6.7–8.2)	7.1 (4.4–9.0)	**0.0396** ^‡^
Lymphocytes (×10^3^/μL)	1.7 (1.2–2.2)	1.6 (1.3–2.1)	1.3 (1.1–1.4)	1.1 (0.9–1.5)	**0.0043** ^‡,¶^
Monocytes (×10^3^/μL)	0.8 (0.7–1.0)	0.7 (0.5–0.8)	0.7 (0.6–0.8)	0.6 (0.4–0.7)	**0.0219** ^†^
Basophils (×10^3^/μL)	0.0 (0.0–0.1)	0.0 (0.0–0.1)	0.0 (0.0–0.0)	0.0 (0.0–0.0)	**0.0277** ^§^
Eosinophils (×10^3^/μL)	0.1 (0.0–0.3)	0.1 (0.1–0.3)	0.1 (0.1–0.1)	0.1 (0.0–0.1)	0.2928
Platelets (×10^3^/μL)	267.5 (229.0–298.0)	253.0 (182.0–320.0)	234.0 (197.0–270.0)	188.5 (138.0–275.0)	0.2736
MPV (fL)	10.4 (9.6–11.4)	10.5 (10.4–11.0)	10.6 (10.2–10.8)	10.5 (10.3–10.9)	0.8939

Results are shown as median ± 95% confidence intervals as analyzed by the Kruskal–Wallis test. ALT, alanine transferase; AST, aspartate transferase; ALP, alkaline phosphatase; GGT, gamma-glutamyl transferase; RBCs, red blood cells; MCH, mean corpuscular hemoglobin; MCHC, mean corpuscular hemoglobin concentration; MCV, mean corpuscular volume; RDW-CV, coefficient of variation in red cell distribution width; WBCs, white blood cells; MPV, mean platelet volume. * Mild vs. non-anemic. ^#^ Moderate vs. non-anemic. ^†^ Severe vs. non-anemic. ^‡^ Mild vs. moderate. ^¶^ Mild vs. severe. ^§^ Moderate vs. severe. Bold *p* values are statistically significant.

**Table 3:** Comparison of inflammatory markers stratified by anemia severity.

Variable	Non-Anemic	Mild	Moderate	Severe	*p*
PNI	42.8 (39.4–48.6)	40.8 (36.4–47.7)	35.8 (33.7–37.2)	32.3 (30.8–34.1)	**<0.0001** ^#,†,‡,¶^
HALP	30.6 (22.6–46.2)	24.0 (20.1–30.7)	14.5 (12.9–16.2)	9.8 (8.0–14.1)	**<0.0001** ^#,†,‡,¶^
R ratio	0.7 (0.6–1.0)	0.5 (0.3–0.8)	0.5 (0.4–0.6)	0.4 (0.3–0.5)	**0.0015** ^#,†^
SIRI	4.5 (2.1–8.0)	1.9 (1.3–2.7)	4.7 (3.4–5.7)	3.5 (2.0–5.6)	**0.0022** *^,‡^
NLR	4.4 (3.3–8.9)	2.5 (2.1–4.8)	6.0 (5.1–7.0)	6.7 (4.2–10.0)	**0.0026** ^‡,¶^
RPR	0.1 (0.0–0.1)	0.1 (0.0–0.1)	0.1 (0.1–0.1)	0.1 (0.1–0.1)	**0.0077** ^†^
LMR	1.7 (1.3–2.4)	2.6 (2.1–3.5)	1.7 (1.5–2.0)	2.0 (1.7–2.5)	**0.0115** ^‡^
PNR	37.6 (24.7–57.9)	52.2 (38.8–70.9)	30.2 (25.0–34.4)	37.7 (24.8–47.6)	**0.0149** ^‡^
FIB-4	1.1 (0.9–1.4)	1.1 (0.8–2.0)	1.5 (1.3–2.0)	2.2 (1.4–3.3)	**0.0166**
AISI	818.9 (397.8–2119.0)	469.7 (256.2–898.9)	889.2 (699.5–1151.0)	627.5 (386.6–1176.0)	**0.0283**
GPR	0.3 (0.1–0.5)	0.1 (0.1–0.2)	0.3 (0.2–0.3)	0.2 (0.2–0.4)	**0.0446** ^‡^
CALLY	6.7 (4.0–16.1)	5.5 (2.4–10.7)	3.8 (2.9–4.8)	2.5 (2.0–3.5)	**0.0472** ^†^
SII	1255.0 (592.1–2028.0)	742.7 (448.7–1124.0)	1339.0 (1014.0–1526.0)	1119.0 (884.1–1910.0)	0.0567
dNLR	2.6 (1.9–4.6)	1.6 (1.2–3.8)	3.3 (3.0–3.6)	3.9 (2.7–5.5)	0.0657
MPVLR	4.9 (4.0–7.6)	5.7 (4.8–7.7)	7.5 (6.5–8.0)	8.3 (6.1–11.2)	0.0677
PCR	3.4 (2.7–4.2)	3.9 (2.2–4.5)	2.1 (1.6–2.8)	1.5 (0.9–2.4)	0.0749
AST APRI	0.3 (0.2–0.4)	0.2 (0.2–0.5)	0.3 (0.3–0.5)	0.5 (0.3–0.8)	0.1181
Platelets/CRP	3.3 (1.7–7.2)	1.6 (1.0–4.6)	2.4 (1.8–3.5)	1.6 (1.0–2.9)	0.1643
CAR	2.4 (0.9–3.4)	3.3 (1.3–7.0)	3.7 (2.6–4.5)	4.7 (2.7–6.1)	0.2112
PMR	305.6 (276.9–364.0)	356.3 (257.1–618.8)	355.0 (310.0–395.0)	405.0 (338.3–467.1)	0.2322
PLR	156.9 (111.3–202.2)	149.5 (116.8–177.6)	180.7 (158.1–206.3)	194.6 (139.7–223.0)	0.2431
Lymphocytes/CRP	0.0 (0.0–0.0)	0.0 (0.0–0.0)	0.0 (0.0–0.0)	0.0 (0.0–0.0)	0.2636
LDH (U/L)	323.0 (192.0–734.0)	365.0 (232.0–2544.0)	285.0 (229.0–346.0)	242.0 (205.0–421.0)	0.3341
ALT APRI	0.3 (0.2–0.4)	0.2 (0.1–0.3)	0.2 (0.2–0.3)	0.2 (0.1–0.3)	0.3516
Lactate (mM)	1.6 (0.8–4.0)	2.9 (1.2–6.6)	1.5 (1.3–2.3)	1.5 (1.0–1.9)	0.4018
Platelets/lymphocytes/CRP	2.2 (1.5–4.2)	1.2 (0.6–4.6)	2.1 (1.4–3.3)	1.8 (1.0–2.6)	0.4114
ELR	0.1 (0.0–0.1)	0.1 (0.0–0.1)	0.1 (0.1–0.1)	0.0 (0.0–0.1)	0.6223
LAR	10.0 (5.6–19.9)	13.2 (8.1–61.5)	10.4 (8.3–12.9)	9.5 (6.9–17.7)	0.6449
CRP (mg/L)	90.1 (29.7–135.6)	89.7 (42.7–240.3)	94.5 (74.1–126.6)	108.9 (76.4–160.6)	0.6636
PAR	7.5 (6.4–8.9)	7.7 (6.4–9.1)	8.5 (7.2–9.4)	6.8 (5.4–10.6)	0.8453
ESR (mm/h)	55.0 (29.0–78.0)	46.0 (21.0–67.0)	55.0 (47.0–65.0)	54.5 (37.0–68.0)	0.9436
Procalcitonin (µg/L)	0.8 (0.2–3.9)	0.4 (0.2–6.4)	0.8 (0.5–1.3)	0.7 (0.3–1.6)	0.9739

Results are shown as median ± 95% confidence intervals as analyzed by the Kruskal–Wallis test. PNI, prognostic nutritional index; HALP, hemoglobin–albumin–lymphocyte–platelet index; SIRI, systemic inflammation response index; NLR, neutrophil–lymphocyte ratio; RPR, RDW-CV–platelet ratio; LMR, lymphocyte–monocyte ratio; PNR, platelet–neutrophil ratio; FIB-4, fibrosis-4 index; AISI, aggregate index of systemic inflammation; GPR, gamma-glutamyl transferase–platelet ratio; CALLY, C-reactive protein–albumin–lymphocyte index; SII, systemic inflammatory index; dNLR, derived neutrophil–lymphocyte ratio; MPVLR, mean platelet volume–lymphocyte ratio; PCR, platelet–creatinine ratio; AST APRI, aspartate transaminase–platelet ratio index; CAR, CRP/albumin ratio; PMR, platelet–monocyte ratio; PLR, platelet–lymphocyte ratio; LDH, lactate dehydrogenase; ALT APRI, alanine transaminase–platelet ratio index; ELR, eosinophil–lymphocyte ratio; LAR, LDH–albumin ratio; PAR, platelet–albumin ratio; ESR, erythrocyte sedimentation rate. * Mild vs. non-anemic. ^#^ Moderate vs. non-anemic. ^†^ Severe vs. non-anemic. ^‡^ Mild vs. moderate. ^¶^ Mild vs. severe. Bold *p* values are statistically significant.

**Table 4:** Correlation of anemia severity with clinical parameters.

Variable	*ρ*	*p* Value	*q* Value
MATH-1SD	0.295	**<0.0001**	**0.0005**
Direct bilirubin	0.269	**<0.0001**	**0.0005**
RDW-CV	0.266	**<0.0001**	**0.0005**
Total bilirubin	0.218	**0.0002**	**0.00088**
RPR	0.183	**0.0015**	**0.00465**
GPR	0.183	**0.0015**	**0.00465**
Hypertension	0.182	**0.0049**	**0.00949**
ALP	0.179	**0.0019**	**0.00535**
*Pseudomonas aeruginosa*	0.177	**0.0022**	**0.00568**
ICU	0.171	**0.0036**	**0.00797**
PO_4_	0.168	**0.0035**	**0.00797**
MPV	0.164	**0.0074**	**0.0124**
Vancomycin trough	0.164	**0.0044**	**0.00909**
FIB-4 index	0.156	**0.0071**	**0.0124**
Creatinine	0.154	**0.0076**	**0.0124**
CRP/albumin ratio	0.145	**0.019**	**0.0245**
Liver disease	0.145	**0.0121**	**0.0187**
AST APRI	0.136	**0.0184**	**0.0245**
Renal disease	0.131	**0.0235**	**0.0291**
GGT	0.126	**0.0291**	**0.0311**
CRP	0.121	**0.0493**	**0.0493**
Platelets	−0.128	**0.0274**	**0.0303**
CALLY	−0.128	**0.0392**	**0.0405**
MCHC	−0.129	**0.025**	**0.0298**
Albumin	−0.137	**0.0182**	**0.0245**
Platelet/lymphocyte/CRP ratio	−0.138	**0.0272**	**0.0303**
Platelet/CRP ratio	−0.150	**0.0155**	**0.0228**
PCR	−0.197	**0.0006**	**0.0023**
Hematocrit	−0.308	**<0.0001**	**0.0005**
RBCs	−0.312	**<0.0001**	**0.0005**
Hemoglobin	−0.327	**<0.0001**	**0.0005**

Spearman’s correlation coefficient (*ρ*) is shown for significant variables ranked by strength and direction of association with anemia severity. False discovery rate-adjusted *p* values (*q* values) are provided to account for multiplicity. RDW-CV, coefficient of variation in red cell distribution width; RPR, RDW-CV–platelet ratio; GPR, gamma-glutamyl transferase–platelet ratio; ALP, alkaline phosphatase; ICU, intensive care unit; MPV, mean platelet volume; FIB-4, fibrosis-4 index; CRP, C-reactive protein; AST APRI, AST-to-platelet ratio index; CALLY, CRP–albumin–lymphocyte index; PCR, platelet–creatinine ratio; RBCs, red blood cells. Bold *p* and *q* values are statistically significant.

**Table 5:** Independent predictors of anemia severity.

Variable	B	Standard Error	95% CI	*p* Value
Univariate				
CALLY	1.388	0.498	0.407 to 2.369	**0.0057**
Lymphocyte/CRP	0.9908	0.489	0.027 to 1.954	**0.0438**
PCR	0.8341	0.351	0.143 to 1.525	**0.0182**
R ratio	0.7742	0.371	0.042 to 1.506	**0.0382**
Platelets	0.5613	0.230	0.1078 to 1.015	**0.0155**
PNI	0.4111	0.064	0.2841 to 0.5382	**<0.0001**
Gender	0.3363	0.150	0.206 to 1.531	**0.0103**
CO_2_	0.1601	0.068	0.0259 to 0.294	**0.0195**
Ca^2+^	0.1165	0.030	0.0567 to 0.176	**0.0002**
RDW-CV	−0.3509	0.048	−0.447 to −0.254	**<0.0001**
MATH-1SD	−0.3747	0.049	−0.472 to −0.276	**<0.0001**
ALP	−0.396	0.188	−0.766 to −0.025	**0.0365**
Renal disease	−0.6559	0.246	−1.141 to −0.170	**0.0083**
MPVLR	−0.7531	0.211	−1.169 to −0.337	**0.0004**
RPR	−0.9123	0.243	−1.391 to −0.433	**0.0002**
AST APRI	−1.006	0.448	−1.888 to −0.123	**0.0257**
FIB-4	−1.135	0.377	−1.878 to −0.392	**0.0028**
ICU	−1.208	0.323	−1.844 to −0.571	**0.0002**
Direct bilirubin	−1.299	0.361	−2.01 to −0.587	**0.0004**
Multivariable				
Albumin	0.325	0.051	0.225 to 0.425	**<0.0001**
Age	0.101	0.031	0.039 to 0.163	**0.0015**
Gender	−0.020	0.009	−0.037 to −0.003	**0.0206**
PNR	−0.023	0.010	−0.043 to −0.003	**0.0198**
Renal disease	−0.034	0.013	−0.060 to −0.007	**0.0108**
ICU	−0.035	0.010	−0.054 to −0.016	**0.0003**
MATH-1SD	−0.336	0.056	−0.446 to −0.226	**<0.0001**

The unstandardized coefficient (B) of each significant predictor is shown as analyzed by simple and multivariable linear regression using log-transformed hemoglobin levels as the dependent variable. The multivariable model is adjusted for liver disease, heart disease, hypertension, diabetes mellitus, coagulopathy, cancer, serum creatinine, vancomycin trough levels, and MRSA infection. Covariates are arranged by strength and direction of association. CRP, C-reactive protein; CALLY, CRP–albumin–lymphocyte index; PCR, platelet/creatinine ratio; PNI, prognostic nutritional index; RDW-CV, coefficient of variation in red cell distribution width; ALP, alkaline phosphatase; MPVLR, mean platelet volume/lymphocyte ratio; RPR, RDW-CV/platelet ratio; AST APRI, AST-to-platelet ratio index; FIB-4, fibrosis-4 index; ICU, intensive care unit. PNR, platelet/neutrophil ratio. Bold *p* values are statistically significant.

## Data Availability

The data that support the findings of this study are available upon reasonable request from the corresponding author (M.A.A.) and with permission from King Saud University.
